# The JAZF1-SUZ12 fusion protein disrupts PRC2 complexes and impairs chromatin repression during human endometrial stromal tumorogenesis

**DOI:** 10.18632/oncotarget.13270

**Published:** 2016-11-10

**Authors:** Xianyong Ma, Jinglan Wang, Jianhui Wang, Charles X. Ma, Xiaobin Gao, Vytas Patriub, Jeffrey L. Sklar

**Affiliations:** ^1^ Department of Pathology, Yale University School of Medicine, New Haven, CT, USA; ^2^ University of Connecticut School of Medicine, Farmington, CT, USA

**Keywords:** SUZ12 and PRC2 complex, endometrial stromal Sarcoma (ESS), t(7, 17) translocation, histone methyl transferase (HMT), H3K27Me3

## Abstract

The Polycomb repressive complex 2 (PRC2), which contains three core proteins EZH2, EED and SUZ12, controls chromatin compaction and transcription repression through trimethylation of lysine 27 on histone 3. The (7;17)(p15;q21) chromosomal translocation present in most cases of endometrial stromal sarcomas (ESSs) results in the in-frame fusion of the JAZF1 and SUZ12 genes. We have investigated whether and how the fusion protein JAZF1-SUZ12 functionally alters PRC2. We found that the fusion protein exists at high levels in ESS containing the t(7;17). Co-transient transfection assay indicated JAZF1-SUZ12 destabilized PRC2 components EZH2 and EED, resulting in decreased histone methyl transferase (HMT) activity, which was confirmed by *in vitro* studies using reconstituted PRC2 and nucleosome array substrates. We also demonstrated the PRC2 containing the fusion protein decreased the binding affinity to target chromatin loci. In addition, we found that trimethylation of H3K27 was decreased in ESS samples with the t(7;17), but there was no detectable change in H3K9 in these tissues. Moreover, re-expression of SUZ12 in Suz12 (−/−) ES cells rescued the neuronal differentiation while the fusion protein failed to restore this function and enhanced cell proliferation. In summary, our studies reveal that JAZF1-SUZ12 fusion protein disrupts the PRC2 complex, abolishes HMT activity and subsequently activates chromatin/genes normally repressed by PRC2. Such dyesfunction of PRC2 inhibits normal neural differentiation of ES cell and increases cell proliferation. Related changes induced by the JAZF-SUZ12 protein in endometrial stromal cells may explain the oncogenic effect of the t(7;17) in ESS.

## INTRODUCTION

Nuclear histone methylation plays a central role in transcription regulation of genes [1–4]. Methylation modification on these histones has been shown to either inhibit or activate gene expression depending on the type of amino acids and the site as well as the degree of methylations [5, 6]. In mammalian cells, the histone methylation level is maintained both by methyltransferase complexes (“Writers”) and demethylase complexes (“Erasers”), the methylation status on histone (histone code) is then recognized by transcription factors or non-coding microRNA molecules (“Readers”), therefore active or inactive target gene expression and play the biological roles [7–9]. Polycomb repressive complexes (PRCs) as the major “writers” are the main players of epigenetic silence. And the silencing status can be transmitted from embryos to adulthood [10, 11]. PRCs were initially identified in Drosophila and later studies showed that the PRC proteins are highly evolutionarily conserved and consist a superfamily. At present there are more than 37 members in mammal have been identified [11, 12]. The different PRC proteins functionally form distinct complexes that belong to two major families of PRC1 and PRC2.

The first purified PRC proteins were those associated with the PRC1 complex of drosophila such as polycomb (PC) [13], polyhomeotic (PH) [14], posterior sex comb (PSC) [15], and dRING [16]. The PRC1 complexes have very diverse composition, all of PRC1 complexes contain Ring 1B (also known as Ring2/RNF2), which has the E3 ubiquitin ligase activity [17, 18], as well as one of the PCGF proteins (PCFG1-6) [19]. Based on PCGF associated PRC1 complexes, Gao et al. classified PCR1 into six subgroups (PRC1.1, PRC1.2, PRC1.3, PRC1.4, PRC1.5, PRC1.6) [20]. Alternatively, a simplified classification for PRC1 is based on presence or absence of chromobox (Cbx) protein, therefore the PRC1 can be classified into cPRC1 and ncPRC1 [20–22]. The canonical function of PRC1 complexes is bond to the site of H3K27Me3 of chromatin therefore form the polycomb repression domain and lead to stabilizing the compacted chromatin [23–26], but *in vivo* study also revealed that variant PRC1 complexes (PHC2, for example) are proficient at catalyzing H2AK119ub1 on chromatin, and surprisingly, this modification auto-polymerizes through its sterile-alpha motif (SAM) [27], and PRC1 can recruit PRC2 to chromatin through recognition of H2AK119ub1 marker, leading to chromatin compaction and gene silencing.

PRC2 is the major class of histone methylation complexes in mammalian cells. PRC2 contains with three core components: SUZ12 (Suppressor of Zest-12 protein) [28]; histone methyltransferase EZH2 (Enhancer of Zeste Homolog 2) [29] and EED (embryonic ectodermal development protein) [30, 31]. These three proteins are presented in a 1:1:1 stoichiometry, and are sufficient for PRC2 function *in vitro* [32]. There are also several variant trimeric complexes due to existence of EZH2 and EED paralogs and splicing isoforms of EZH2 and EED. It has been identified that the PRC2-EZH2 mediates gene repression via catalyzing methylation of H3K27 [33, 34], but the function of PRC2-EZH1 remains large unknown. A number of PRC2 cofactors have been identified that modify the PRC2 activity and recruitment, such as Rbap46/48; AEBP2; Sir T1; HDAC (NAD+- dependent histone deacetylase; Jarid2; PCL1 (PHF1); PCL2 (MTF2); PCL3 (Phf19); C17orf96 and C10orf12 [35–37]. Furthermore, the recently findings indicate long noncoding (Such as Malat1, Rajaram V. et al.) [38] RNAs also involve in the activity regulation of PRC2. The varied activities of PRC2 can produce from allosteric effect of these cofactors or partners. Therefore PCR2 functionally catalyzes core histone methylation and initiates compaction of targeted chromatin regions (PRC Response Elements, PRE) [39, 40].

PRC2 and its components have recently been associated with carcinogenesis and metastasis. For example, EZH2 increases in several human tumors, such as Hodgkin lymphoma [41], prostate and breast cancers [42, 43]. Upregulation of EZH2 expression is also associated with poor prognosis and is a feature of metastatic cancers [44–46]. It has been characterized that cytoplasmic function of EZH2-associated methyltransferase polymerization through regulation of GTP binding activity is involved in adhesion and migration capabilities [47, 48], which may affect metastasis ability of malignant cells. *In vitro* studies demonstrate that EED protein differs in the length of their N termini, which governs the histone substrate specificity of PRC2 binding complexes, and is involved in the formation of transformation-specific complexes [49]. Direct evidence also shows EED and SUZ12 lost in malignant peripheral nerve sheath tumors and recurrently inactivated PRC2 activity [50]. Down-regulation of SUZ12 expression is reported to associate with HBV-induced liver carcinogenesis [51]. Chromosome abnormalities involving polycomb proteins have been frequently detected in human endometrial stromal sarcoma (ESS) patients, In low grade ESS, the most frequent genetic rearrangement is the t(7;17)(p15;q21) [52], which results in genetic fusion of JAZF1 and SUZ12, which was originally referred to as JJAZ1. That the chromosomal rearrangements are closely associated with women's ESSs indicate these genetic events may play critical role in carcinogenesis/ metastasis. Unfortunately, until to date the biochemical/pathological function of the fusion proteins derived from gene rearrangements in ESS tumors remain large unclear.

The genetic rearrangement of JAZF1 with SUZ12 genes produces chimeric fusion protein JAZF1-SUZ12. The JAZF1 is a nuclear factor, which represses the transcription process via the interaction with nuclear orphan receptor TR4 [53]. The SUZ12 is the most recently identified component of the PRC2 complex, this protein contains a Zn-finger domain and a VEFS [VER2-EMF2-FIS2-Su (z) 12] box, which is conserved in putative plant homologs EMF2, VERN2 and FIS2. Suz12 (−/−) ES cells are impaired in proper differentiation, resulting in lack of repression of ES cell factors via globally loss of H3K27 trimethylation [54]. Other experiments have also shown that SUZ12 plays a role in cell cycle and X chromosome inactivation [55]. Our previously work has shown the JAZF1-SUZ12 fusion causes allelic exclusion and therefore the unrearranged SUZ12 allelic is suppressed [56]. Suz12 (−/−) ES cells are impaired in lack of repression of stemness factors via globally loss of H3K27 trimethylation, and therefore are damaged in proper differentiation [54]. Other experiments have also shown that SUZ12 plays a role in cell cycle and X chromosome inactivation [55]. To explore the biochemical role of fusion protein JAZF1-SUZ12 (gain of function mutation) in ESS carcinogenesis, we carried out *ex vivo* and *in vitro* experiments to explore the structural and functional consequences of this fusion on the PRC2 comples, including interactions with other components of PRC2, binding activities with target chromatin, and methyltrasferase activities on target histones. We also performed functional assays for fusion protein using Suz12 (−/−) knockout ES cells. Based on these experiment results, we found that the fusion protein JAZF1-SUZ12 recurrent inactivated the PCR2 activity cells cultured *in vitro*. We proposed a model to elucidate the role of fusion protein JAZF1-SUZ12 on ESS carcinogenesis.

## RESULTS

### The t(7;17) translocation results in production of the JAZF1-SUZ12 fusion protein in human ESS

**Figure 1:**
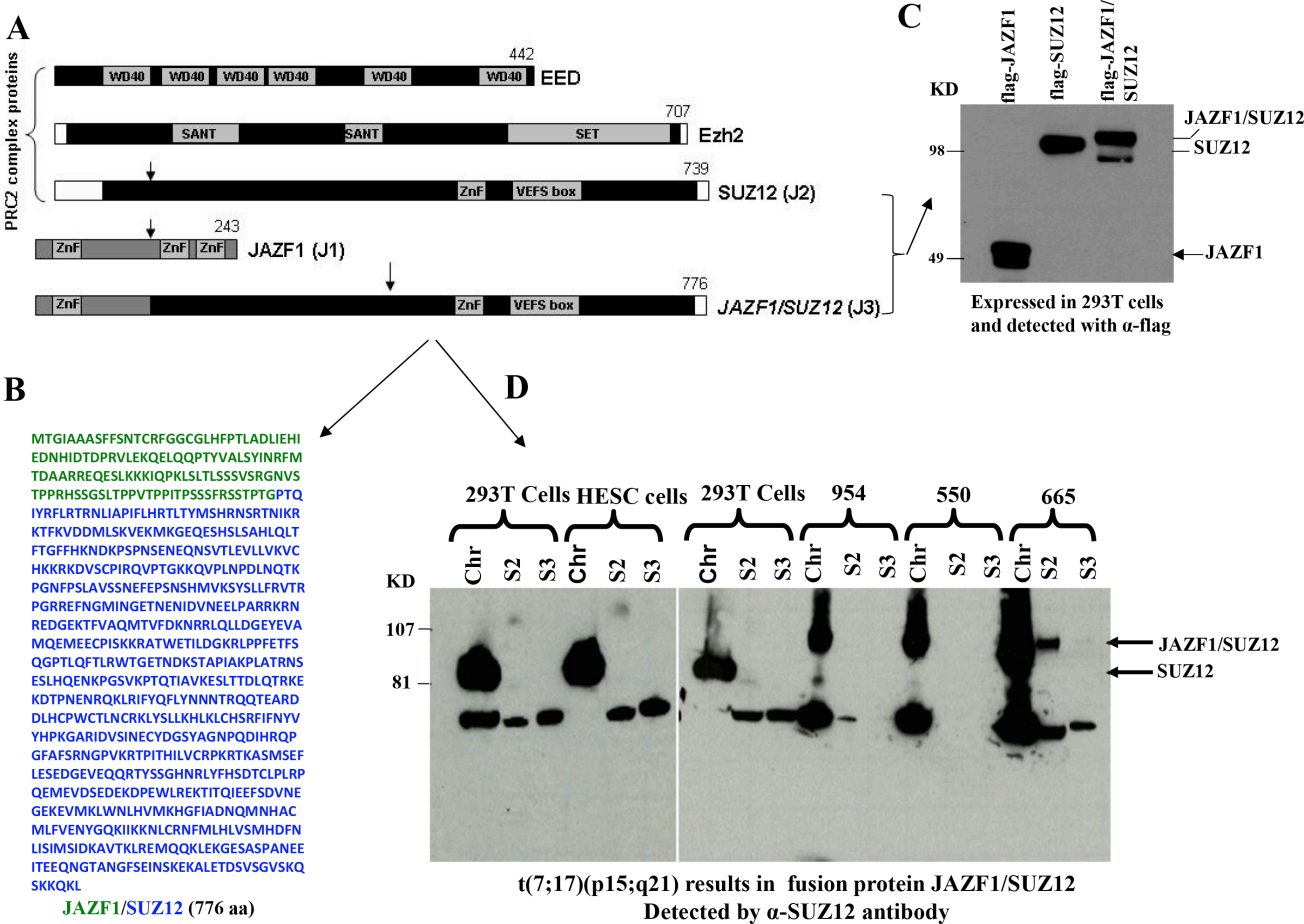
Domain structure of PRC2 components, sequence of fusion protein JAZF1-SUZ12 and its expression in human endometrial stromal sarcoma harboring the t(7;17) (**A**). Schematic drawing representing the functional domains of EED, EZH2, SUZ12 and the fusion protein JAZF1-SUZ12. A portion of JAZF1 containing an N-terminal zinc finger domain is fused to the bulk of SUZ12 in the JAZF1-SUZ12 protein. (**B**). The amino acid sequence of fusion protein JAZF1-SUZ12, labeled in blue color to indicate the sequence derived from JAZF1, and green from SUZ12. (**C**). Western blot showing the flag tagged JAZF1, SUZ12 and fusion protein JAZF1-SUZ12 expressed in 293T cells. (**D**). Western blot showing the fusion protein present in subcellular fractions from human endometrial stromal sarcoma cells containing t(7; 17). Wild type SUZ12 protein expressed in 293T and HESC cells serves as a control (Chr: chromatin fraction, S2: Soluble cytoplasm fraction, S3: soluble nuclear fraction).

We began our study by detecting JAZF1-SUZ12 fusion protein from endometrial stromal sarcoma patients harboring the (7;1 7) translocation. Previous studies have shown that the most frequent chromosome abnormalities in endometrial stromal sarcoma is a recurrent t(7;17) (p15;q21), leading to an in-frame chimeric RNA joining transcribed from portions of two Zinc finger genes, JAZF1 and SUZ12 (JJAZ1). Here we identified the fusion protein by western blot analysis. Wild type JAZF1 and SUZ12 contain 243 aa and 739 aa respectively as showed in Figure 1A. Based on the position of the breakpoints in the t(7;17), 129 N-termini amino acids from JAZF1 is predicted to replace the 92 N-termini amino acids of wild type SUZ12 and generate a fusion protein JAZF1-SUZ12 that contains the 776 amino acids (Figure 1B). The flag labeled JAZF1, SUZ12 and JAZF1-SUZ12 were expressed in 293T cells as shown in Figure 1C. High level of fusion protein was confirmed from ESS tumor samples as shown in Figure 1D using anti-SUZ12 antibody (and anti-JAZF1 antibody, data not showed).

### JAZF1-SUZ12 fusion protein destabilizes EZH2 and EED

**Figure 2:**
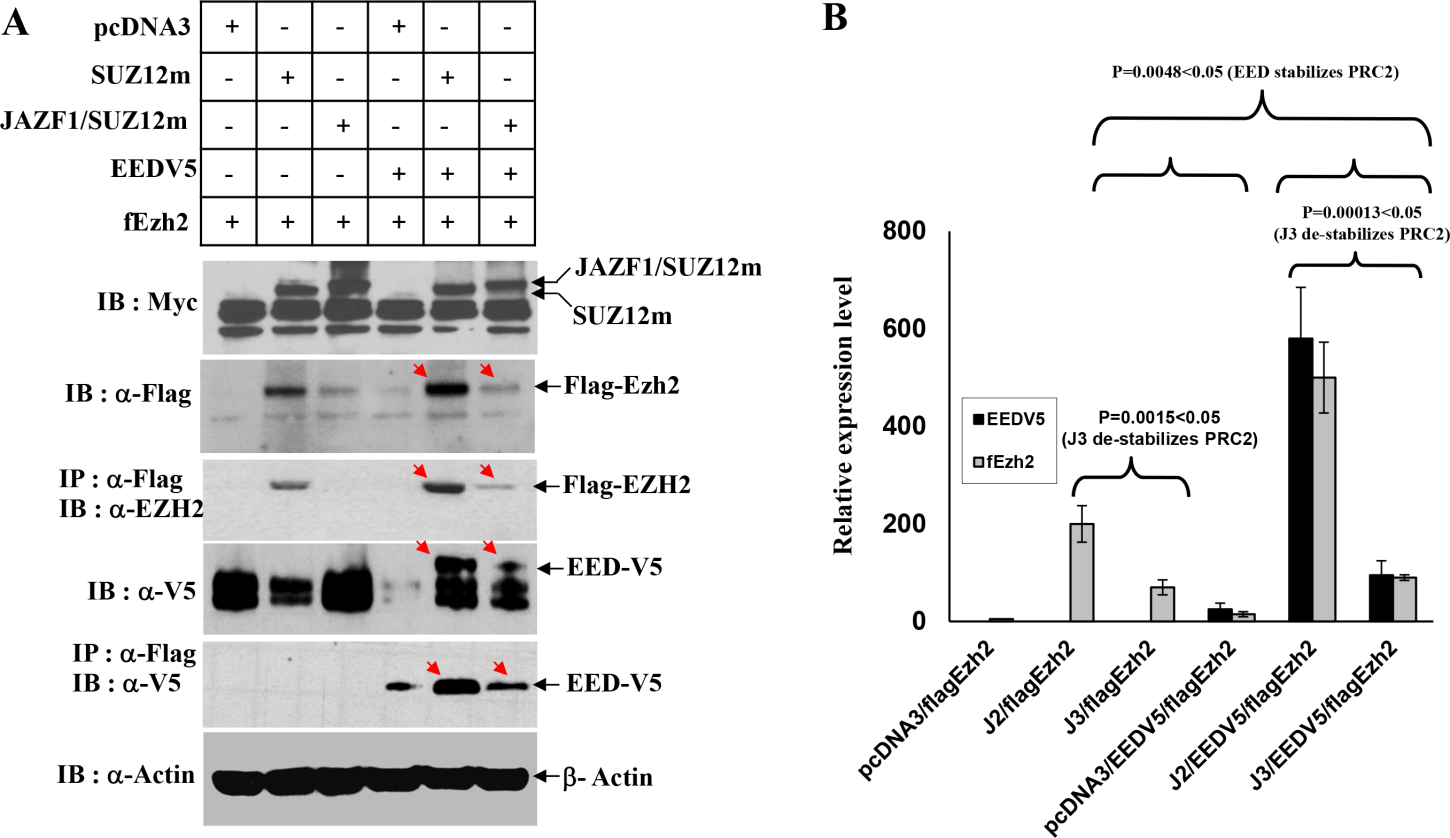
Interactions of JAZF1-SUZ12 protein with EZH2 and EED using co-expression and co-immunoprecipitation (**A**) Western blot for the components of PRC2 complex in extracts of 293T cells co-transfected with vectors encoding epitope-tagged SUZ12myc or JAZF1-SUZ12myc with flagEZH2 and EEDV5. Co-immunopreciptations were carried out with anti-flag agarose, and then blotted with the indicated antibodies. (**B**) The comparison of EZH2 and EED protein levels in different expression experiments. Three different biological repetitions were performed to measure the signal intensity of target bands by densitometry, and the results used to calculate the mean ± SD or the significance analysis. (J2 = SUZ12, J3 = JAZF1-SUZ12).

To identify if JAZF1-SUZ12 fusion protein changes HMT activity of PRC2, we first performed a transiently cotransfected experiment and expression of the exogenesis SUZ12, fusion protein JAZF1-SUZ12, EZH2, and EED were detected by Co-IP and western blot analysis (Figure 2A). In the presence of comparable amounts of SUZ12 and JAZF1-SUZ12, EZH2 and EED were much lower in abundance in cells expressing JAZF1-SUZ12, while levels of AEBP2 and RBAP48 were not significantly different (data is not shown) in cells bearing expression vectors for SUZ12 and JAZF1-SUZ12. These results are consistent with that of JAZF1-SUZ12 significantly decreasing EZH2 and EED protein levels, in the presence or absence of EED (Figure 2B). In contrast, two other Polycomb protein RBAP48 and AEBP2 levels were not affected by the replacement of SUZ12 with JAZF1-SUZ12.

### JAZF1 fusion with SUZ12 prevents the co-localization of SUZ12 with EZH2 and EED, and abolishes the HMT activity of the PRC2 complex

**Figure 3:**
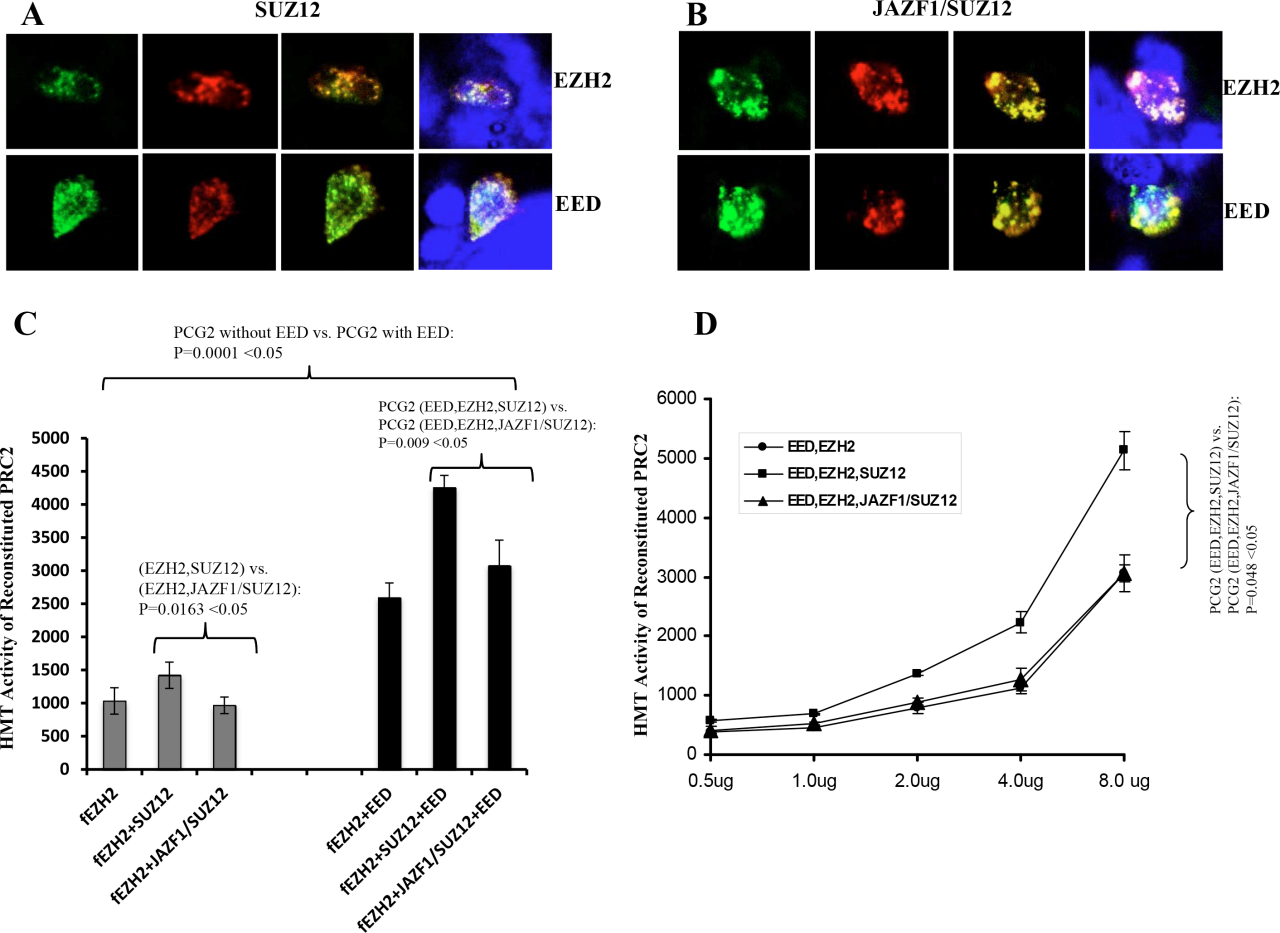
Fusion of JAZF1 with SUZ12 decreases co-localization with EZH2 and EED, and lowers the HMT activity of PRC2 (**A**) Wild type SUZ12 co-localized with EZH2 (upper panel) and EED (bottom panel). (**B**) JAZF1-SUZ12 co-localized with EZH2 (upper panel) and EED (bottom panel). The colors of red and green were from direct fluorescence of RFP-EZH2 or RFP-EED and GFP-SUZ12 or GFP-JAZF1-SUZ12 respectively. Blue color represents nuclear DNA stained with DAPI. The average overlap of SUZ12 with EED and EZH2 are 25–30% higher than that of fusion protein JAZF1-SUZ12 with EED and EZH2. (**C**) Assay of the HMT activity of PRC2 complexes with SUZ12 or with JAZF1-SUZ12, which were purified by immunoprecipitation using α-flag antibody. [3H]-labeled S-adenosylmethionine and purified histones from Hela cells were used as the substrates. Three repetitions of these analyses were used to measure the HMT activity of the PRC2 complexes, the significant difference between fusion protein group and wild type SUZ12 group was determined as in Figure 2. (**D**) Assays of the HMT activity with varying concentrations of purified PRC2 complexes.

To verify the subcellular interactions, we co-transiently transfected 293T cells with GPP-SUZ12 or GFP-JAZF1-SUZ12 expression vectors in combination with RFP-EZH2 or RFP-EED vectors. Confocal imaging revealed punctuated patterns of fluorescent proteins of GFP-SUZ12, GFP-JAZF1-SUZ12, RFP-EED and RFP-EZH2 in peripheral sections of the nucleus (Figure 3A and 3B). The green fluorescence dots of SUZ12 and JAZF1-SUZ12 were distributed in perichromatin region together with red fluorescence dots of EZH2 or EED. The co-localization was measured by counting the overlapping dots of red and green fluorescent proteins. The analysis showed wild type SUZ12 around 86% co-localized with EZH2 and 80% with EED, but fusion protein JAZF1-SUZ12 around 56% with EZH2 and 50% with EED. These results showed that SUZ12 fusing with transcriptional factor JAZF1 reduces the binding of the fusion SUZ12 with EZH2 and EED in the nucleus. Analysis of HMT activities showed that PRC2 (with or without EED) in the presence of JAZF1-SUZ12 is significantly decreased compared to that in the presence of SUZ12. EED significantly enhanced HMT activity of PRC2 complex (Figure 3C). The dose dependent HMT activity was confirmed by first order kinetics with respect to cell lysates when the substrate histone concentration was the same for each reaction (Figure 3D).

### HMT activity detection of PRC2 complexes contains SUZ12 or fusion protein JAZF1-SUZ12

**Figure 4:**
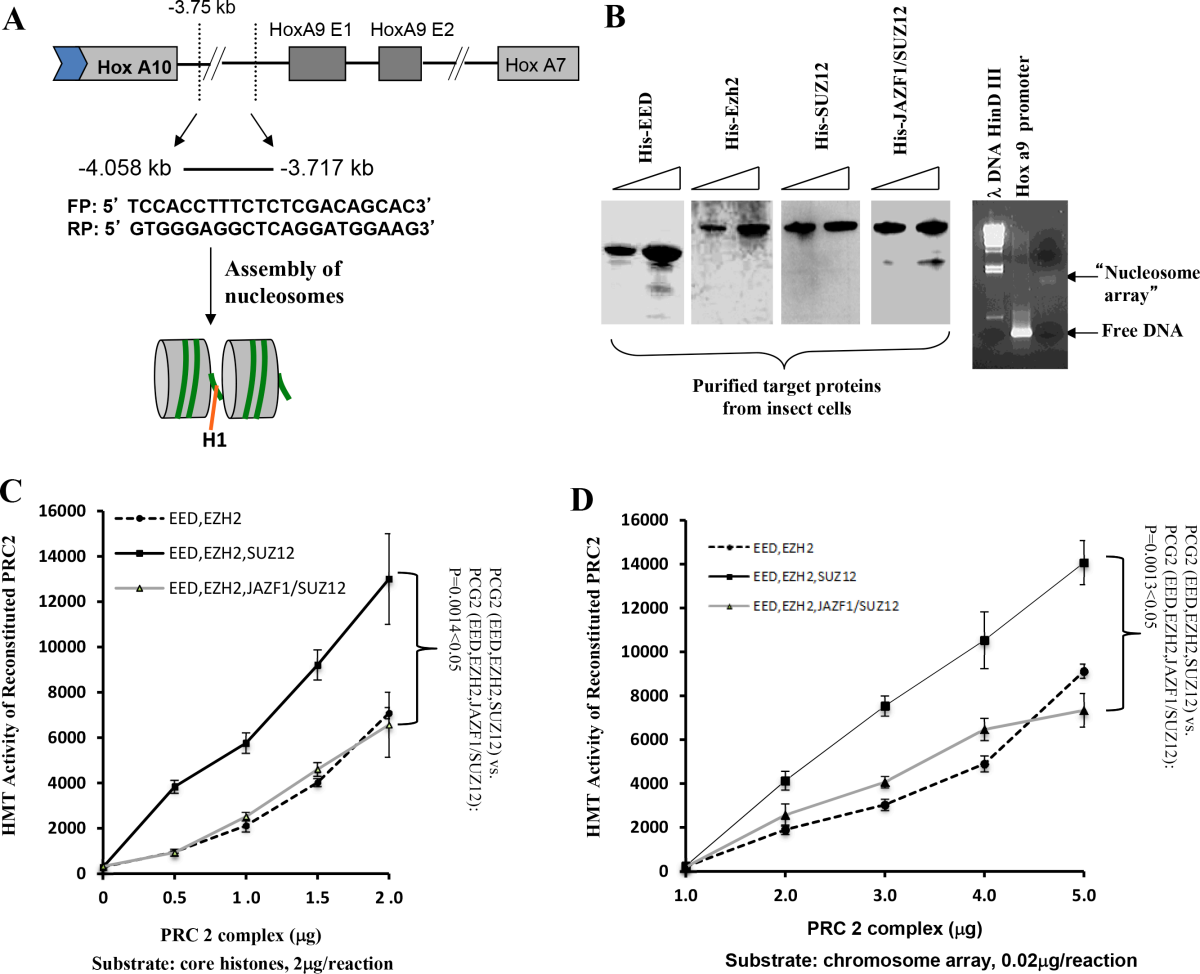
The level of HMT activity of reconstituted PRC2 complexes assayed with nucleosome arrays containing the HOXA9 promoter depends upon the form of SUZ12 in the complexes (**A**) Schematic drawing representing the HOXA9 gene showing two exons and a 341-basepair region spanning the transcriptional start site to which PRC2 binds. DNA from this region was amplified used the primers shown in the figure and the product was incubated with histones purified from HeLa cells to assemble chromatin potentially containing two nucleosome arrays, as illustrated in the gel image stained with ethidium bromide (B, right panel). (**B**) Analysis of epitope-tagged PRC2 components (His-EZH2, His-EED, His-SUZ12, and His-JAZF1-SUZ12) produced in high 5 insect cells and purified using Ni-NTA agarose beads. Protein bands were visualized by Coomassie brilliant blue stain of a polyacrylamide gel (upper panel) or by western blot (lower panel). (**C**) Assessment of HMT activity using [3H] SAM, 2 μg of core histones as substrate, and 0 to 2 μg of purified PRC2 proteins from insect cells. (**D**) Similar to (C), using 0.02 μg of reconstituted nucleosome array as the substrate. Three measurements were performed to determine the difference between complexes with wild type SUZ12 and with JAZF1-SUZ12.

To confirm the observation that JAZF1-SUZ12 reduces HMT activity, we carried out an *in vitro* experiment using reconstituted nucleosome arrays as substrate. As shown in Figure 4A, a 340bp HOXA9 promoter fragment, which is a PRC2 target sequences [18], was used to construct the nucleosome arrays with histone proteins derived from chicken red cells. The *in vitro* assembled nucleosome arrays were checked by agarose gel (Figure 4B, right). His-tagged EZH2, EED, SUZ12 and JAZF1-SUZ12 produced separately in High 5 insect cells and purified using Ni-agarose beads were visualized using commasie brilliant stain (Figure 4B, left). Purified proteins of EZH2, EED and SUZ12 or EZH2, EED and JAZF1-SUZ12 were mixed together in proximately stoichiometrical molar amounts in the presence of 4M urea. Assembly was then initiated by dialysis against reconstituted buffer RCB (see Material and Methods), and the reconstituted PRC2 or its derivatives were then used for *in vitro* HMT assays. We then performed HMT assays using core histones as substrate. The results indicated the HMT activity of PRC2 with fusion protein is around 50% of the PRC2 with wild type SUZ12 using the same amounts of core histone substrates—a difference that was significant (*P* < 0.05) over three independent assays, as demonstrated in Figure 4C. Using a substrate of core histones in reconstituted nucleosome arrays, the HMT activity of wild type PRC2 or PRC2 with the fusion protein was increased around 100-fold (Figure 4D). This experiment also showed that the JAZF1-SUZ12 fusion protein not only partially disrupted PRC2 and its components, but it also interfered with the methyltransferase activity of the complex.

### JAZF1-SUZ12 reduces binding activity of PRC2 to chromatin and releases repression of target genes *in vitro*

**Figure 5:**
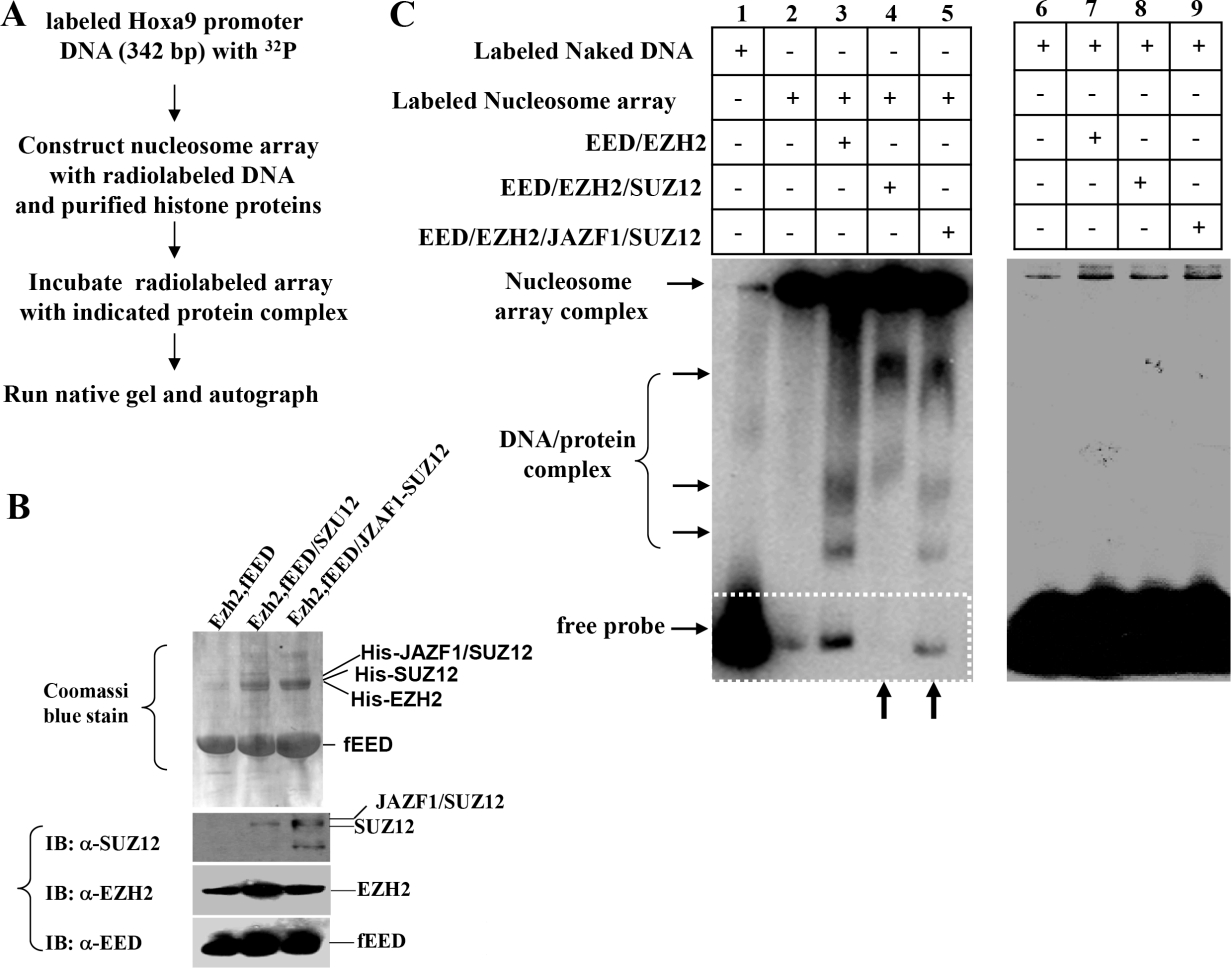
Fusion protein JAZF1-SUZ12 disrupts the interaction of PRC2 to the nucleosome array of target gene promoter *in vitro* (**A**) Schematic representing the procedure used to detect the effect of JAZF1-SUZ12 protein on the interaction of PRC2 complexes with nucleosome arrays of HOXA9 DNA described in Figure 4. PRC2 proteins were purified from insect cells with Ni-NTA agarose and equal amount of proteins were used for each reaction. (**B**) Detected PRC2 complex proteins EED, EZH2, SUZ12 and JAZF1-SUZ12 in co-purified complexes by Coomassi brilliant blue stain and Western blot. (**C**) Electrophoretic mobility shift assay (EMSA) showing the effect of PRC2 complexes with wild type SUZ12 or JAZF1-SUZ12. Left panel: EMSA of 32P-labeled nucleosome array with three different PRC2 complexes. The PRC2 complex components in the incubation are shown at the top of the gel (lanes 1–5). Right panel: EMSA of 32P-labeled naked DNA fragment as a control.

Since PRC2 is a critical chromatin repression complex, it is important to test if JAZF1-SUZ12 affects the binding of PRC2 complex with target chromatin. We first designed a chromatin electrophoretic mobility shift assay (EMSA) as shown in Figure 5A. PRC2 complexes including EZH2/flagEED, SUZ12/EZH2/ flagEED, JAZF1-SUZ12/ EZH2/flagEED were prepared separately in co-expressed insect cells and then purified with anti-flag agarose beads. Purified PRC2 complexes were electrophoretically separated on SDS-PAGE gels (Figure 5B, upper) and individual components were checked by western blot (Figure 5B, lower). ^32^P-labeled 342 bp DNA fragments containing the HOXA9 promoter and commercial histones were used for reconstitution of nucleosome arrays. These arrays were incubated with purified PRC2 complexes, run on 6% native gel and finally visualized by autoradiography (Figure 5C, left panel). The naked DNA fragments labeled with radioactivity were used as the EMSA control (Figure 5C, right panel). The results showed that naked DNA shifted as free probe on lane 1 of left panel, the radio labeled nucleosome arrays stayed in the wells due to their large molecular weight; very small amount of free probe was detected from this well (Figure 5C, left panel, lane 2). When incubated with EZH2/EED complex, the labeled array released some free DNA probes (Figure 5C, left panel, lane 3), while incubated with EED/EZH2/SUZ12 complex, the labeled array did not release any free probes (Figure 5C, left panel, lane 4). In contrast, the PRC2 contained JAZF1-SUZ12 fusion protein showed significant detached probes compared with PRC2 contained SUZ12, (Figure 5C, left panel, lane 5). The 342 bp naked DNA probe was also incubated with reconstituted PRC2 complexes, no shift bands were detected in the reactions (Figure 5C, right panel).

**Figure 6:**
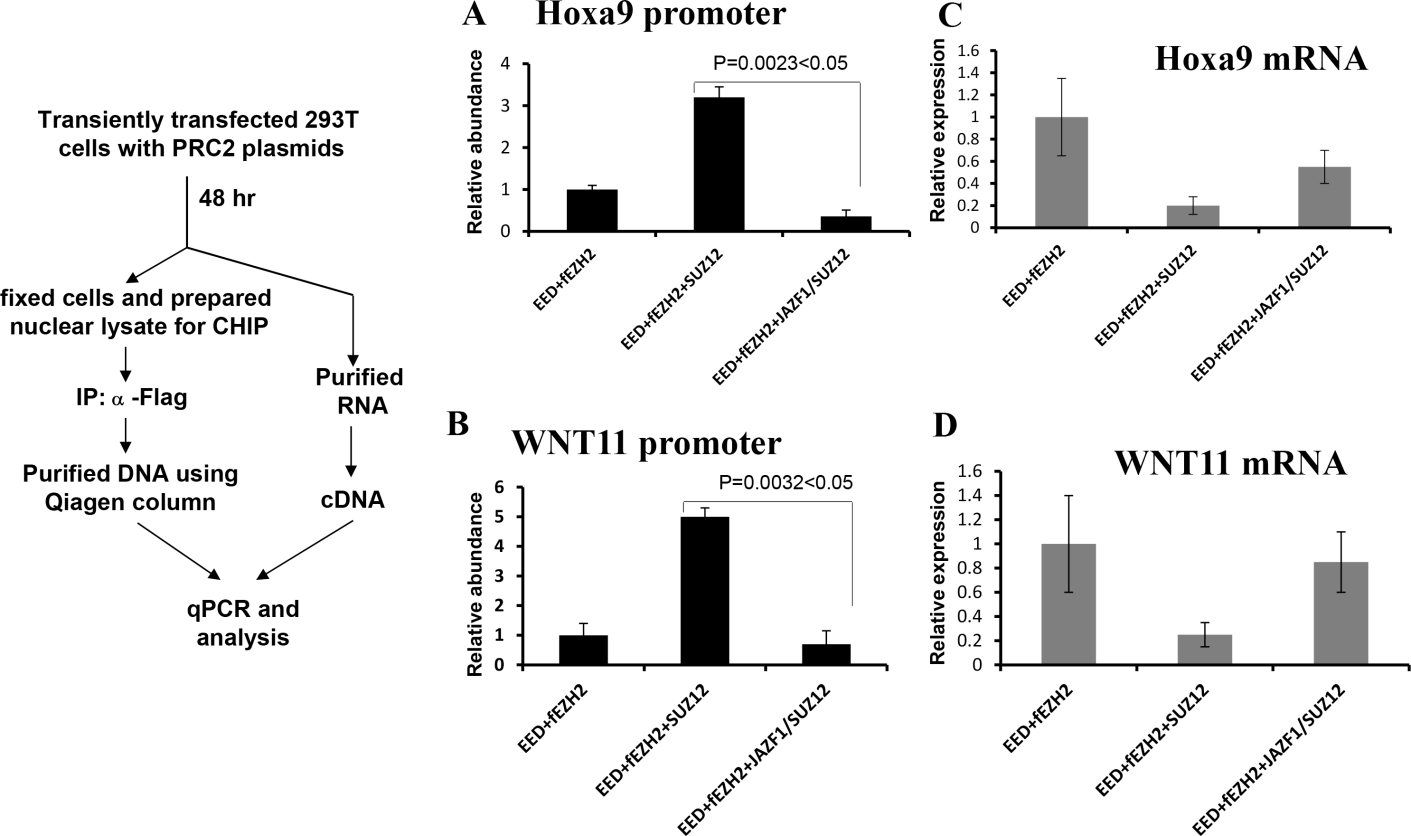
Fusion protein JAZF-SUZ12 reduces the binding activity of PRC2 with target promoters and enhances its expression Left panel: Schematic representation of the procedure used to detect the effect of JAZF-SUZ12 on binding to chromatin promoters. (**A**) JAZF-SUZ12 decreased the binding activity (9-fold) of PRC2 complexes with HOXA9 promoter DNA. (**B**) JAZF-SUZ12 decreased the binding (7 fold) of PRC2 complex to WNT11 promoter. Relative abundance of promoter DNA was determined by quantitative PCR in three independent experiments. (**C**) JAZF1-SUZ12 reduced the PRC2 inhibition to Hoxa9 expression and the mRNA significantly increased. (**D**) JAZF1-SUZ12 reduced the PRC2 inhibition to WNT11 expression and the mRNA significantly increased.

To confirm the above *in vitro* result, we carried out the *ex vivo* chromatin immunoprecipitation (CHIP) assays to detect promoter-binding activities of PRC2 with wild type SUZ12 or with fusion protein JAZF1-SUZ12. Figure 6A and 6B show results of CHIP for *ex vivo* binding activities with HOXA9 and WNT11 promoter regions In chromatin. The binding activities of PRC2 contained SUZ12 bound with HOXA9 or WNT11 promoter regions were 4–7 times higher than that of PRC2 containing JAZF1-SUZ12 fusion protein. Furthermore, the expression analysis for these two genes showed wild type PRC2 complex significantly decreased HOXa9 and WNT11 expression level, but PRC2 with fusion protein JAZF1-SUZ12 showed the much less inhibition to the expression of these two genes (Figure 6C and 6D).

### JAZF1-SUZ12 interferes with ES cell differentiation and enhances proliferation

**Figure 7:**
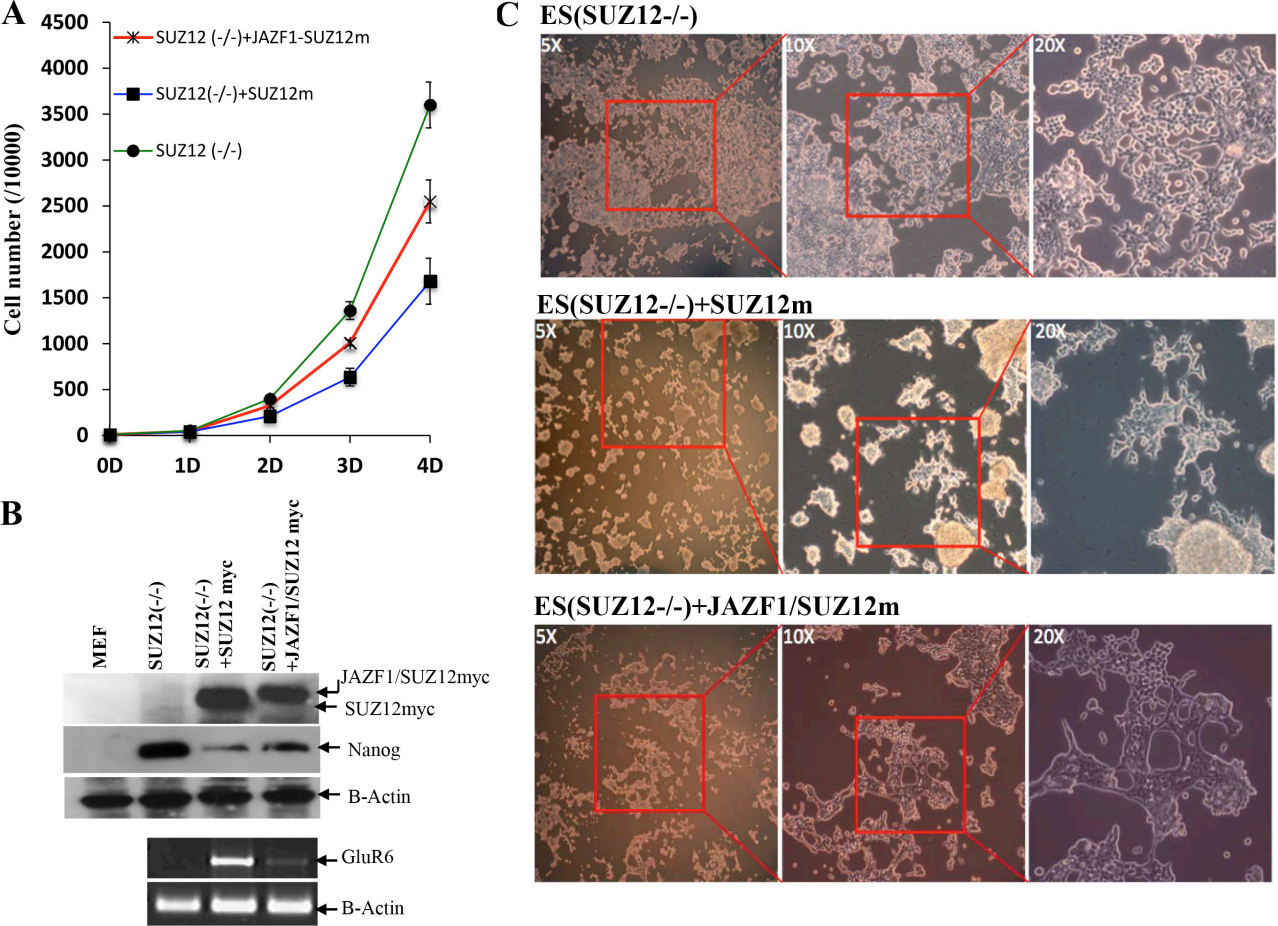
Expression of fusion protein JAZF1-SUZ12 does not rescue the neuronal differentiation of Suz12 (−/−) ES cells (**A**) Proliferation rate of three cell lines of Suz12 (−/−), Suz12 (−/−) +SUZ12 and Suz12 (−/−) +JAZF1-SUZ12. The counting was started from the beginning of EB induction with ATRA treatment [25]. (**B**) Upper panel: Western blot analysis of the ES cell marker protein NANOG in ES cells expressing SUZ12 or JAZF1-SUZ12, after treatment with ATRA, Bottom panel: RT-PCR to detect expression of the neuronal marker RNA GLUR6 in differentiated ES cells. (**C**) Phase contrast pictures of neuronal differentiation cells. Upper panel: Suz12 (−/−) ES cells, middle panel: Suz12 (−/−) +SUZ12, bottom panel: Suz12 (−/−) +JAZF1-SUZ12.

To determine if the fusion protein changes normal biological function with respect to cell proliferation and differentiation, we expressed SUZ12 or JAZF1-SUZ12 from plasmids in murine Suz12 (−/−) ES cells. EB body cells that were either Suz12 (−/−), Suz12 (−/−) +SUZ12, or Suz12 (−/−) +JAZF1-SUZ12 derived from ES cell were induced with all trans-retinoic acid (ATRA), as described in the methods and materials. As shown in Figure 7A, the proliferation rates of the three cell lines were significantly different after day 4 of ATRA treatment. The Suz12 (−/−) ES cells expressing SUZ12 had lower proliferation rates than Suz12 (−/−) ES cells expressing JAZF1-SUZ12. Both Suz12 (−/−) ES cells expressing either SUZ12 or JAZF1-SUZ12 proliferated slower compared to Suz12 (−/−) ES cells, and expression of the ES cell marker Nanog mRNA was reduced around 3 fold in the cells of expressing SUZ12 or the fusion protein. During differentiation assays, the neuron-like differentiation was found in Suz12 (−/−) +SUZ12 cells (more than 50% of cells), but was absent in Suz12 (−/−) and Suz12 (−/−) +JAZF1-SUZ12 cells (Figure 7A). PCR analysis indicated the neuronal-specific marker GLUR6 was significantly activated in Suz12 (−/−) + SUZ12 cells. A faint band from Suz12 (−/−) +JAZF1-SUZ12 cells indicated weak activation of the Glu46 gene in these cells.

### JAZF1-SUZ12 reduces methyltransferase activity of PRC2 and decreases trimethylated H3K27 in ESS tumor samples

**Figure 8:**
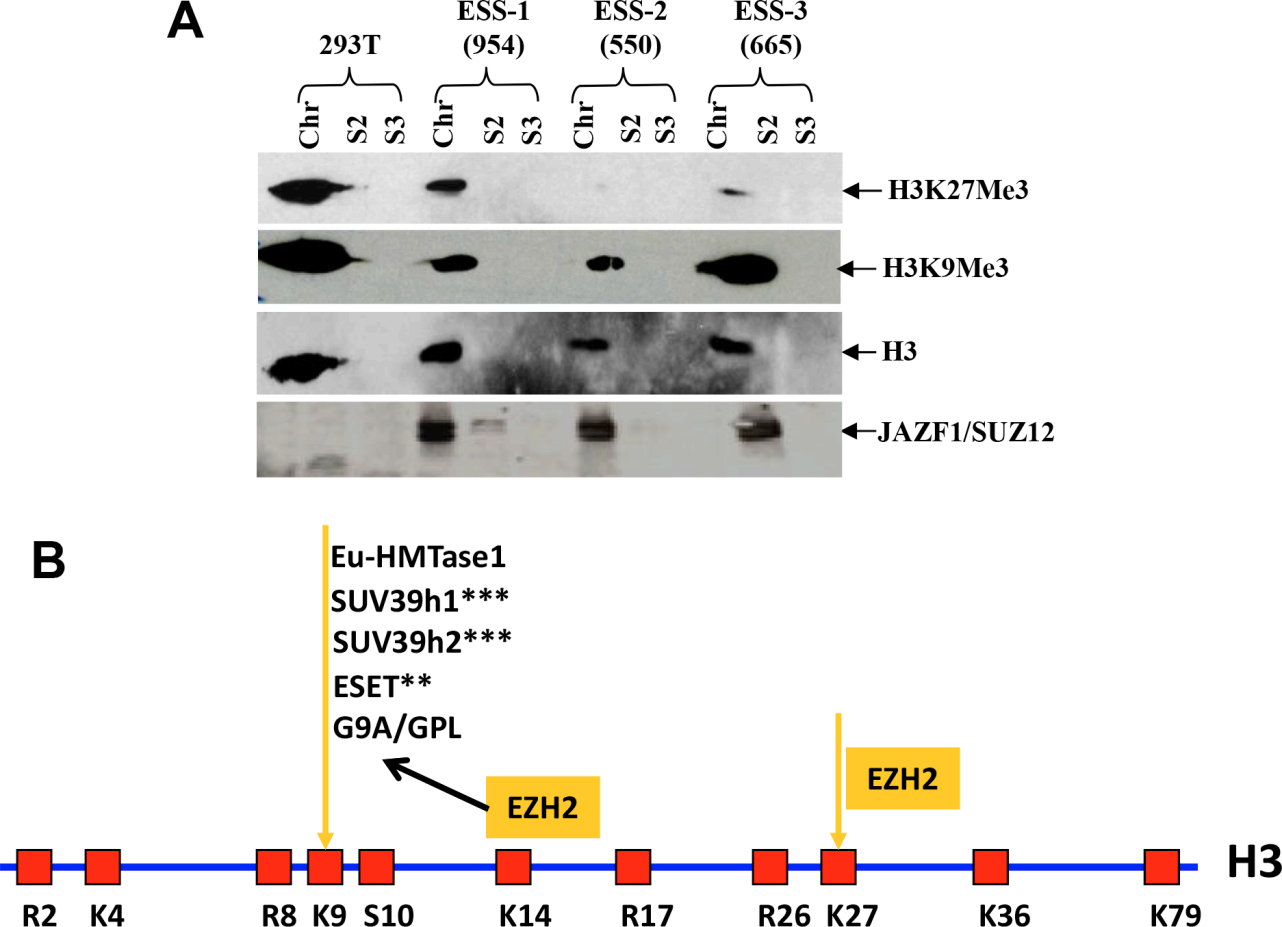
t(7;17) and JAZF1-SUZ12 expression in human ESS dramatically decreases H3K27 trimethylation (**A**) Top panel: H3K27me3 in different ESS tumor samples contained JAZF1-SUZ12; second panel: H3K9me3 level in different ESS tumor samples; third panel: core histones as the loading control. JAZF1-SUZ12 was detected using anti-JAZF1 antibody. (Chr: chromatin fraction; S2; soluble cytoplasm fraction; S3: soluble nuclear fraction.) (**B**) Schematic representation of the arginine (R) and lysine (K) residues in the core histone 3. K9 and K27 are the methylation target sites of the PRC2 complex.

Based upon *in vitro* and *ex vivo* studies, we concluded that the fusion protein JAZF1-SUZ12 is at least partially disruption of PRC2 and leads to lower amounts of core components EZH2 and EED, thereby reducing methyltransferase activity of the PRC2 complex and decreasing binding of target promoters (HOXA9 and WNT11). To investigate whether ESS tumors containing the t(7;17) and JAZF1-SUZ12 expression have abnormal H3K27me3 levels, we analyzed human endometrial stromal sarcoma cells having the fusion protein JAZF1-SUZ12 by western blot (Figure 8B). The result showed that the level of H3K27me3 was markedly reduced in cells derived from three ESS cases. Notably there was no detectable H3K27me3 for case 550# (Figure 5). Comparatively, JAZF1-SUZ12 does not change the level of H3K9me3, which normally catalyzes by other complex.

## DISCUSSION

Human endometrial stromal sarcomas (ESS) represent relatively rare tumors (< 10% of all malignant uterine tumors). In large part because of their rarity, not much has been known at a molecular level about oncogenic events underlying the occurrence of these neoplasms. However, a series of recurrent, nonrandom cytogenetic abnormalities on chromosome 1, 6, 7, 10, 17 and X have been identified in ESSs. Among these chromosomal abnormalities, t(7;17)(p15;q21), which results in an in frame fusion of the *Jazf1* gene with *Suz12* on chromosome 17 is the most common cytogenetic abnormalities [52]. Other translocations include the t(6;7)(p21;p15), which results in an in-frame fusion of *Jazf1* to *Phf1* [59]; the t(6;10)(p21;p11), which results in an *Epc1* fused to *Phf1* [58]; the t(1;6)(p34;p21), which results in an in-frame fusion of *Meaf6* gene to *Phf1* [59]; der(22)(X;22)(p11;q13) results in *Zc3h7b* in-frame joints to *Bcor* gene [60]; t(X;17)(p11;q21) results in *Mbtd1* gene fuses to *Cxorf67* [61]. If the fusion of in-frame remains unknown; t(10;17)(q22;p13) results in *Ywhae* gene in-frame joins to *Fam22a* or *Fam22b*; this translocation is also known as 14-3-3 epsilon-FAM22A (FAM22B) [62]. Some unidentified chromosome translocation in ESS have been reported such as der (3) t(3;6)(q29;p21.1), t(X;17)(p11;q23) [63, 64]. The chromosome rearrangements have been used as one of the important indicators of the WHO classification and clinical diagnosis of ESS [65]. For example, JAZF1-SUZ12 fusion exists predominantly in endometrial nodule and low grade ESS, and the YWHAE-FAM22 fusion is associated within high grade and undifferentiated ESS. Other translocations and fusions were only identified from low grade ESS.

The pathological significances of these chromosome abnormalities and resulting fusion proteins in ESS carcinogenesis are still largely unknown. To date, evidence consistent with a gene fusion resulting in the oncogenic protein in ESS comes from research on the YWHAE-FAM22. Silence of *Ywhae-Fam22* expression reversed the malignant phenotype of ESS tumor cells harboring this fusion gene, as indicated by the reduction of the proliferation rate and cell migration [66]. Therefore this fusion protein acts as an oncoprotein during ESS carcinogenesis. YWHAE involves in cell metabolism, protein trafficking, signal transduction and cell apoptosis. The function of FAM22A/B protein is still unclear. Of all the fusion proteins associated with ESS, the YWHAE-FAM22 is the only one not known to involve proteins that perform chromatin epigenetic modifications. Although it is unclear how this protein causes ESS carcinogenesis, the relocation of YWHAE from cytoplasm to nucleus by fusion with FAM22 probably demonstrates that the oncoprotein obtains some function for chromatin modification and regulation of gene expression. More experiments are needed to address this question

A striking feature concerning the molecular genetics of ESSs is that, among the seven chromosomal translocations identified within them, six are associated with chromatin modifications that derived from the fusion proteins JAZF1-SUZ12, JAZF1-PHF1, EPC1-PHF1, MEAF6-PHF1, MBTD1-CXorf67 and ZC3H7B-BCOR. SUZ12, PHF1 and MBTD1 belonging to Polycomb proteins involve in core histone methylations. BCOR protein is a component of a corepressor complex that represses methylation of H3K4 and H3K36 [67].

**Figure 9:**
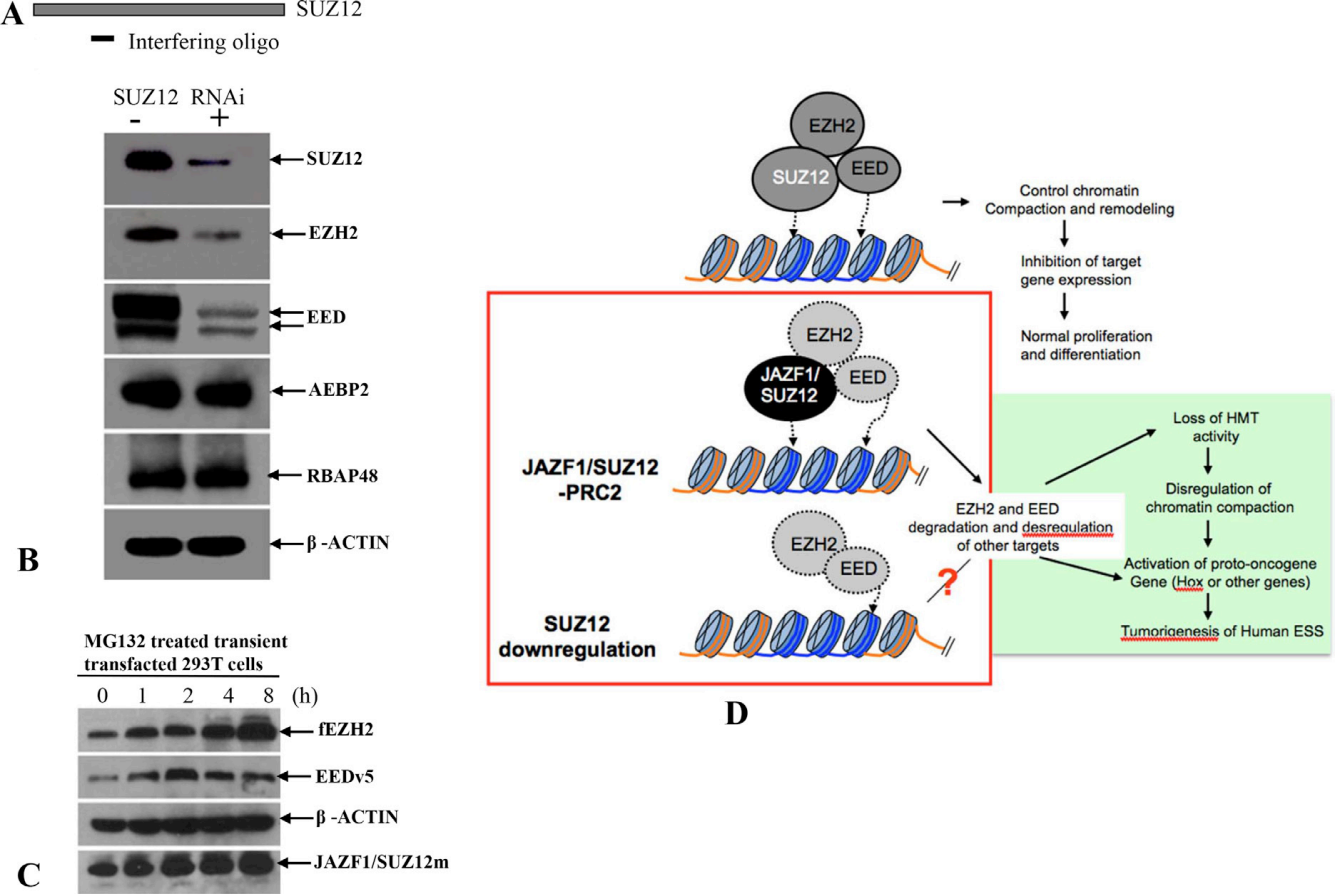
A model of the relationship between impairment of chromatin repression by JAZF1-SUZ12 and ESS tumorigenesis (**A**) RNA interference oligonucleotide complementary to sequence near the 5′terminal of SUZ12 mRNA. (**B)** Western blot analysis of SUZ12, which was knockdown in 293T cells. The target proteins were indicated in the figure. β-actin was used as the loading control. (**C**) Western blot analysis showing the accumulations of EZH2 and EED in 293T cells co-expressing EZH2, EED and JAZF1-SUZ12, and treated with the proteasome inhibitor MG132. (**D**) A model shows the relationship of JAZF1-SUZ12, PRC2 function, and ESS tumorigenesis in human endometrial stromal cells.

The research described here was intended to elucidate processes within the cells that are affected by the presence of the JAZF1-SUZ12 fusion. This research showed that the fusion protein reduces the methyltransferase activity of the PRC2 complex via destabilization of this complex and its core components EED and EZH2. In addition the proteasome inhibition assay demonstrated MG132 treatment can increase the levels of these two proteins (Figure 9C). We conclude that JAZF1-SUZ12 destabilizes PRC2 through a proteasome-dependent mechanism. The reduction of the SUZ12 protein level in cells by RNA interference resulted in destabilization of EED and EZH2 (Figure 9A and 9B). These findings are consistent with *ex vivo* SUZ12 concentration being important for stabilization of PRC2 components. In addition, our study shows that SUZ12 fused with JAZF1 not only destabilizes PRC2 and reduces methyltransferase activity, but also directly impedes the enzymatic activity of PRC2 complex (Figure 4C, 4D). We conclude that both the structure and concentration by SUZ12 protein greatly affects PRC2 function via stabilization of EED and EZH2. Efficient H3K27 methylation of PRC2 requires wild-type structure of SUZ12 and proper cytoplasm concentration of this protein.

The relationships among core histone methylation, chromatin modifications and gene expression are well established. Generally, H3K4me3, H3K36me3 and H3K79me3 are associated with chromatin decompaction and gene activation. In contrast, H3K9me3, H3K20me3 and H3K27me3 are responsible for chromatin compaction and gene repression. The degree of methylation at all of these lysine residues correlates with transcriptional activity. Recent studies reveal that mono-methylation at the residues of H3K9, H3K27 and H4K20 was mostly distributed within transcriptionally active chromatin regions and linked with gene activation [68, 69]. The JAZF1-SUZ12 fusion protein leads to predominantly reduction of the H3K27Me3 level but doesn't change the H3K9Me3 level catalyzed by G9A/GPL, which is dependent on EZH2 recruitment [70]. This result suggests that the PRC2 of JAZF1-SUZ12 specifically decreases H3K27Me3 but H3K9Me3, the H3K9 methylation maybe catalyzed by other PRC2-EZH2 independent enzymes such as ESET, SUV39h1/2 and Eu-HMTase1 when PRC2 activities are reduced by JAZF1-SUZ12 in ESS cells (Figure 8B). More experiments are needed to show the role of feedback regulation on H3K9 methylation in JAZF1-SUZ12 or SUZ12 inhibited cells.

Alterations or abnormalities in histone methylation appear to be important factors in inducing or maintaining the neoplastic phenotype of cancer cells. Modified PRC2 activity seems to be responsible for a sizeable fraction of such changes. In some instance, increased methylation at specific histone amino acids by PCR2 activates oncogenic pathways. For instance, in breast cancer, raised levels of PRC2 leads to high levels H3K27 methylation in the promoter regions of FOXC1, E-cadherin, RAD51, RUNX3 and CDKN1C (p57kip2) [71]. Consequent decreases of FOXC1, E-cadherin and RUNX3 result in enhanced cell invasion and metastasis, therefore PRC2-H3K27 methylation plays an oncogenic role. EZH2 is also activated in many cancers, UTX, an H3K27me3 demethylase, is inactivated in multiple myeloma, esophageal, renal and bladder and in other cancers, the demethylase JMJD3 is expressed at very low levels therefore leads high level of H3K27me3 [72]. On the other hand, low level of EZH2 or reduced methylation at histone sites catalyzed by PRC2 may be oncogenic, for example, the recurrent Tyr 641 mutations in catalytic EZH2 SET domain, thought to impair activity, occur in two types of lymphomas arising from germinal center B cells [73]. Loss of EZH2 in HSCs (hematopoietic stem cells) is sufficient to cause aggressive T-acute lymphoblastic leukemia (T-ALL) in mice [74]. Recently research supports that activation of *Myc* oncogene is positively correlated with decreased PRC2 activities and H3K27me3 level in prostate cancer. That impaired of PRC2 activity by mutation of EZH2 results in a dramatically reduction of H3K27me3 and subsequently promotion of breast tumorigenesis [75].

In this study, we showed that the human endometrial stromal sarcoma with the JAZF1-SUZ12 fusion disrupts PRC2 and decreases H3K27me3. Furthermore, expressed SUZ12 in *Suz12* (−/−) knockout ES cells showed wild type SUZ12 protein can rescue neuronal differentiation function after ATRA treatment, but the fusion protein failed to rescue this differentiation. Therefore our study supported PRC2-SUZ12 effectively inducing ES cell differentiation, that knockout of SUZ12 or mutation of SUZ12 (fusion of SUZ112 with JAZF1) results in blocking of ES differentiation. The analysis of growth in SUZ12 knockout cells showed that the JAZF1-SUZ12 fusion protein expressed in SUZ12 knockout cells also had higher proliferation rates. Taken together, our experiments prove that PRC2/H3K27me3 possess a tumor suppressor function. The PRC2 with JAZF1-SUZ12 profoundly loses suppressor function and acts as an oncogenic-like protein involved in tumorigenesis of human endometrial sarcoma (Figure 9D). Our study highlights that dysfunction of PRC2 as a possible mechanism in human endometrial carcinogenesis and the potential value of PRC2 as a therapeutic target in ESS patients.

## MATERIALS AND METHODS

### Constructs

Full-length cDNA of human JAZF1, SUZ12 and JAZF1-SUZ12 were cloned into pcDNA3.1/NT-GFP-TOPO to generate in-frame fused GFP-SUZ12 and GFP-JAZF1-SUZ12 vectors. Human EZH2 cDNA was cloned into Bam HI site of pDsRed1-N1 to generate RFP-EZH2 vector that fused N- terminal red fluorescent protein (RFP) in-frame to EZH2. Human EED cDNA was cloned into Eco RI site of pDsRed1-N1 to generate in frame fusion vector RFP-EED. pcDNA-flagEZH2 (a kind gift from Yi Zhang), pcDNA-SUZ12myc and pcDNA-JAZF1-SUZ12myc were described previously [22]. Human EED, RBAP48 (a kind gift from Patty Wendel) and AEBP2 (cloned from human epithelia cells) were cloned into pcDNA3.1D/V5-His-TOPO to generate in frame fusion vectors pcDNA3.1 /EEDV5, pcDNA3.1-RBAP48 and pcDNA3.1-AEBP2. Full length human EZH2 cDNA fragments containing Bam HI overhangs on both sites was ligated into the Bam HI site of plasmid pFast Bac (A), full length EED, SUZ12 and JAZF1-SUZ12 cDNA fragments containing Eco RI overhangs on both sites were ligated into the Eco RI site of plasmid pFast Bac (A). HOXA9 promoter DNA was isolated by PCR amplification using the DNA from human Hela cells, as showed in Figure 4A. The structures of all constructs were confirmed both by restriction enzymatic digestion and DNA sequencing.

### Cell culture and stable cell lines

HEK 293T cells were cultured in DMEM medium (GIBCO). Human endometrial stromal cells (HESC) were maintained in DMEM/F12 (GIBCO). The media were supplemented with 10% fetal bovine serum, 100 U of penicillin and 100 μg of streptomycin per ml (Invitrogen), 10 mM sodium pyruvate and 5.5 mM glutamine, Cells were cultured at 37°C in 5% CO2 incubator. Initial ESS tumor cells were from Prof. Sklar lab. [58]. Briefly, three low-grade ESS cells were identified with t(7;17)(p15;q21). For case 665 (BWH-665) was from a metastatic vaginal mass in a 69-yr-old woman, for cases 550 (Lu-550) and 965 (LU-965) were both from primary uterine tumors in 41-yr-old women. Primary ESS tumor cells were amplified in RPMI 1640 supplemented with 20% FCS under standard conditions.

### Purification of PRC2 proteins and prepared nucleosome array

High 5 insect cells were infected with the indicated viruses produced from SF21 using the Bac-to-Bac baculovirus system following the manufacturer's protocol (Invitrogen). Coomassie blue stain of the gels or western blot were used to test the purified proteins. Assembly of nucleosome arrays was carried out following previously described methods [62]. *In vitro* reconstituted PRC2 complexes as following: purified proteins of EZH2, EED and SUZ12 or EZH2, EED and JAZF1-SUZ12 were mixed together in approximately the same amounts in the presence of 4 M urea, and dialyzed against reconstituted buffer RCB (see below). Assembled array and naked DNAs were resolved on 1% agarose gel with 1XTAE buffer.

### *In vitro* methylation assays

Whole cell extracts derived from transfected HEK 293T cells were prepared following manufacturer's instruction (Upstate), and 6 mg portions of extracts was immunoprecipitated overnight at 4°C with the α-flag or α-myc antibodies. HMT activity was detected using HMT assay reagent kit, and core histone proteins or reconstituted nucleosomes as substrates Immunoprecipitated proteins were probed for HMT activity by transfer of radiolabeled methyl groups from S- [methyl-3H] AdoMet to the histone substrate, and transfer of the products to phosphocellulose paper. After extensive washed, measuring the 3H radioactivity was measured by scintillation counting.

### Antibodies, immunoprecipitations (IP) and western blots

Rabbit antibodies against EZH2, histone 3, ubiquitin, H3K9me3 and H3K27me3 were purchased from Upstate Company. Rabbit antibody against EED was a kind gift from Dr. Armin Schumacher. Monoclonal anti-His antibody was purchased from GE Company. Mouse anti-actin antibody was purchased from Chemicon Company. Mouse anti-flag antibody, mouse anti-myc antibody, HRP-conjugated anti-rabbit or anti-mouse IgG antibodies were purchased from Sigma Company. Rabbit anti V5, rabbit anti AEBP2, rabbit anti GluR6, rabbit anti Rbap48 were purchased from Abcam Company. Rabbit anti Nanog antibody purchased from Cell Signaling Inc. Rabbit anti-JAZF1 and SUZ12 antiserum were prepared described [58] and further affinity-purified using protein A sepharose columns before use. Immunoprecipitation was carried out following manufacture's protocol (Upstate). For western blot analysis, proteins were separated by SDS-PAGE gel electrophoresis, transferred to PVDF membranes, and detected with the indicated antibodies.

### Microscopy and fluorescent imaging

HEK 293 T cells in two-well chamber slides were transfected or cotransfected with 0.5 μg of pNTGFP, pNTGFP-JAZF1, pNTGFP-JJAZ1, pNTGFP-JAZF1-SUZ12, pRFP-EED and pRFP-EZH2 plasmids using Lipofectamine 2000 (Invitrogen). After 24 h, fluorescence within the cells was assessed by confocal microscopy.

### ChIP analysis and mRNA expression detection

ChIP assay was performed using chromatin immunoprecipitation assay kit (Upstate) following the manufacturer's instructions. HEK 293T cells (2 × 10^6^) were plated in 100-mm dish and transfected with a total 24 μg of indicated plasmids. Cells were treated with 1% formaldehyde at 37°C for 10 min, followed by preparation nuclear extracts and sonication of chromatin DNA into fragments of lengths around 200 bp. Chip assays were carried out with anti-flag antibody, and immunoprecipitated DNA fragments were subjected to quantitative PCR using QuantiTect SYBR Green PCR master mix (Qiagen). The primers for CHIP analysis were as following: HOXA9-F 5′ TCCACCTTTCTC TCGACAGCAC 3′, HOXA9-R 5′ GT GGGAGGCTCAGGATGGAAG 3′; WNT11-F: 5′ TTCC GATG CTCCTATGAAGG 3′, WNT11-R: 5′ AGACACC CCATGGCACTTAC3′. The primers for expression analysis were as following: Hoxa9-Fe: 5′ TCTCGGGGATGC ATAGATTC 3′, Hoxa9-Re: 5′ CTGTTCGTCTGGTGC AAAAA 3′; WNT11-Fe:5′TGACCTCAAGACCCGATA CC 3′, WNT11-Re:5′ GCCCACCTTCTCATTCTTCA 3′; GAPDH-Fe: 5′ CGCTCTCTGCTCCTCCTGTT 3′, GAPDH-Re: 5′ CCATGGTGTCTGAGCGATGT 3′ .

### Electrophoretic mobility shift assay (EMSA) of chromatin

*In vitro* constituted nucleosome arrays containing radiolabed human HOXA9 promoter fragments (as previously described) were incubated with the indicated PRC2 complexes in 20 μl of EMSA buffer [10 mM Tris-HCl, pH 8.0, 1 mM EDTA, 1 mM dithiothreitol, 50 mM NaCl, 1.0 μg poly (dI-dC) per reaction and 5% glycerol] for 30 min on ice, and the mixture was then fractionated by electrophoresis on a 5% non-denaturing polyacrylamide gel. Dried gels were analyzed using a phosphorimager (Fuji Photofilm, image Gauge Version 2.53). For competition experiments, varied amounts of cold competitors were pre-incubated with samples before addition of radiolabeled probe.

### Proteosome inhibition experiments

To examine if inhibition of proteasomes leads to accumulation of EZH2 and EED proteins in transiently transfected cells, 293T cells co-transfected with indicated plasmids were grown for 24 hours, followed by treatment with 50 mM MG132 for different intervals as indicated in figure legends. Lysates were prepared from the cells and proteins detected by western blot analysis.

### Suz12 (−/−) ES cell culture and differentiation induction by ATRA

Mouse Suz12 (−/−) ES cells were a kindly gift from Dr. Diego Pasini. All of ES cells were maintained on mitomycin C (Sigma) treated PMEF cells (mouse embryonic fibroblasts, Stem Cell Technologies) in DMEM medium supplemented with 15% inactivated fetal serum, 0.1 mM nonessential amino acids (GIBCO/BRL), 2 mM glutamine, 50 units/ml penicillin, 50 μg/ml streptomycin (GIBCO/BRL), 0.1 mM 2-mercaptoethanol (Sigma) and 1,000 units/ml leukemia inhibitory factor (Stem Cell Technologies). Suz12 (−/−) ES cells transiently transfected with the expression construct of SUZ12myc or fusion gene JAZF1-SUZ12myc were selected with G418 to establish stable ES cell line expressed SUZ12 or JAZF1-SUZ12. EB body formation was previously described [27]. EB bodies were treated with 0.5 uM all-trans retinoic acid (ATRA), and the cell number counted from day 0 to day 4 to assay the proliferation rate. Day 4 EB bodies were evaluated for the expression of stem cell marker protein NANOG. At day 7, the EB body cells were plated on gelatin-coated dishes to allow ES cell differentiation. Neuronal-specific differentiation of treated cells was assessed by RT-PCR using the following primers: GLUR6-F: 5′ CCAAGATAGAATATGGA GCAGTAGAGG 3′, GLUR6-R: 5′ ACTGTCTCCTGCTGCTCA TAAATG 3′

### RNA interferences (RNAi)

SUZ12 expression in 293T cells was silenced by RNA interference. The sequence corresponding to the target-specific siRNA duplex is underlined as following: 5′ GATCCGACATGGGAGACTA TTCTTGATGGGAA TTCAAGAGATTCCCAT CAAGAATAGTCT CCCATG CTTTTTTACGCGT 3′ (shRNA-Oligo I, corresponding to nucleotides 1235 to 1259 bp of full length human SUZ12 mRNA sequence (Ref. Seq.: NM_015355.3). The oligomers were inserted into retroviral vector RNAi-pSIREN-RetroQ, which drives shRNA production from U6 promoter (Clontech). The inserts were confirmed by DNA sequencing, after which retroviral constructs were transiently transfected into PT67 amphotropic packaging cells using Lipofectamine 2000 (Invitrogen), and the viral supernatants were collected at 24 to 48 h after transfection and stored at −80°C for future use.

### Statistical analysis

For all data analysis, the mean values and standard deviations were calculated from three independent experiments. *P* values were calculated with a paired student's *T* test (two tailed hypothesis) for assessing statistical significance of observed differences. A *P* value of < 0.05 between experimental samples (group) and control samples (group) was considered to be statistically significance.

## Abbreviations

ESS: endometrial stromal sarcoma
PRC2: polycomb repressive complex 2
HMT: histone methyltransferase
HESC: human endometrial stromal cells
EB: embryonic body; fusion protein JAZF1/SUZ12 = JAZF1-SUZ12.
